# Spatiotemporal analysis of suicide attempts in Colombia from 2018 to 2020

**DOI:** 10.1590/0102-311XEN119323

**Published:** 2024-09-16

**Authors:** Mario Julian Cañon-Ayala, Yury Estefania Perdomo-Jurado, Angela Gissette Caro-Delgado

**Affiliations:** 1 Politécnico Grancolombiano, Bogotá, Colombia.; 2 Universidad Nacional de Colombia, Bogotá, Colombia.

**Keywords:** Attempted Suicide, Suicidal Ideation, Mental Health, Bayes Theorem, Intento de Suicidio, Ideación Suicida, Salud Mental, Teorema de Bayes, Tentativa de Suicídio, Ideação Suicida, Saúde Mental, Teorema de Bayes

## Abstract

Suicide is one of the leading death causes worldwide, mainly among young adults, and Colombia has experienced an increase during the XXI century. The suicide impact has diverged between age groups and locations in Colombia, where young adults have taken higher incidences than the other age groups. The COVID-19 lockdown induced changes in mental health, affecting the previous suicide trends in the country. We conducted a spatiotemporal analysis of suicide attempts in Colombia per age group, adopting Bayesian models that represent 85,526 individual records in 1,121 municipalities from 2018 to 2020 using R-INLA. We found that Colombia exhibited an increase in suicide-attempt incidence from 2018 to 2019, and suddenly, the incidence fell in the first semester of 2020. The fixed effect of the models evidenced the highest risk in overall municipalities per trimester in the age group between 15-19 years old. The spatial random effect per model evidenced municipalities with the highest risk in the age groups between 10 to 59 years, mainly in the states in the Andean region of Colombia, and other states such as Putumayo, Vaupés, Arauca, Córdoba, Amazonas, and Meta. The temporal random effect evidenced a decay in suicide trends from the fourth trimester of 2019 to 2020, except in the age group > 59 years old. Geographically, our study pinpointed specific regions in Colombia, particularly in the central, southwest, and southeast areas, where the incidence of suicide attempts exceeded 100 cases per 100,000 inhabitants. The nuanced breakdown of incidence across different age groups further underscores the importance of tailoring preventive strategies based on age-specific and regional risk factors.

## Abstract (es)

El suicidio es una de las principales causas de muerte a nivel mundial, principalmente entre adultos jóvenes, y Colombia ha experimentado un aumento durante el siglo XXI. El impacto del suicidio ha divergido entre grupos de edad y ubicaciones en Colombia, donde los adultos jóvenes han tenido mayores incidencias que los otros grupos de edad. El confinamiento por la COVID-19 indujo cambios en la salud mental, afectando las tendencias suicidas anteriores en el país. Realizamos un análisis espacio-temporal de los intentos de suicidio en Colombia por grupo de edad, adoptando modelos bayesianos que representan 85.526 registros individuales en 1.121 municipios de 2018 a 2020 utilizando R-INLA. Encontramos que Colombia exhibió un aumento en la incidencia de intentos de suicidio de 2018 a 2019, y repentinamente, la incidencia cayó en el primer semestre de 2020. El efecto fijo de los modelos evidenció el mayor riesgo en el total de municipios por trimestre en el grupo de edad entre 15-19 años. El efecto aleatorio espacial por modelo evidenció los municipios con mayor riesgo en los grupos de edad entre 10 a 59 años, principalmente en los estados de la región andina de Colombia, y otros estados como Putumayo, Vaupés, Arauca, Córdoba, Amazonas y Meta. El efecto aleatorio temporal evidenció una disminución en las tendencias suicidas del cuarto trimestre de 2019 a 2020, excepto en el grupo etario > 59 años. Geográficamente, nuestro estudio identificó regiones específicas de Colombia, particularmente en las zonas central, suroeste y sureste, donde la incidencia de intentos de suicidio superó los 100 casos por 100.000 habitantes. El desglose matizado de la incidencia entre diferentes grupos de edad subraya aún más la importancia de adaptar las estrategias preventivas en función de factores de riesgo regionales y específicos de la edad.

## Abstract (pt)

O suicídio é uma das principais causas de morte em todo o mundo, principalmente entre adultos jovens, e a Colômbia experimentou um aumento durante o século XXI. O impacto do suicídio divergiu entre grupos etários e locais na Colômbia, onde os adultos jovens tiveram incidências mais elevadas do que os outros grupos etários. O confinamento da COVID-19 induziu mudanças na saúde mental, afetando as tendências anteriores de suicídio no país. Realizamos uma análise espaço-temporal das tentativas de suicídio na Colômbia por faixa etária, adotando modelos bayesianos que representam 85.526 registros individuais em 1.121 municípios de 2018 a 2020 usando R-INLA. Descobrimos que a Colômbia apresentou um aumento na incidência de tentativas de suicídio de 2018 a 2019 e, repentinamente, a incidência caiu no primeiro semestre de 2020. O efeito fixo dos modelos evidenciou o maior risco no geral dos municípios por trimestre na faixa etária entre 15-19 anos. O efeito aleatório espacial por modelo evidenciou municípios com maior risco nas faixas etárias entre 10 a 59 anos, principalmente na região andina da Colômbia e em outros estados como Putumayo, Vaupés, Arauca, Córdoba, Amazonas e Meta. O efeito aleatório temporal evidenciou queda nas tendências de suicídio do quarto trimestre de 2019 para 2020, exceto na faixa etária > 59 anos. Geograficamente, nosso estudo identificou regiões específicas da Colômbia, particularmente nas áreas central, sudoeste e sudeste, onde a incidência de tentativas de suicídio ultrapassou 100 casos por 100 mil habitantes. A repartição matizada da incidência entre diferentes grupos etários sublinha ainda mais a importância de adaptar estratégias preventivas com base em factores de risco regionais e específicos da idade.

## Introduction

The World Health Organization estimates that 703,000 people worldwide died by suicide in 2019 ^1^. Suicide has become one of the most common causes of death, accounting for one in 100 deaths, and it is the fourth leading cause of death among people aged 19 to 29, after road traffic injuries, tuberculosis, and interpersonal violence. Although Colombia reported 29,792 suicide attempts in 2021, this suicide rate is lower than that found in regions such as North America and Europe, but the country has seen an increase in suicide attempts since 2015 ^2^ ^,^ ^3^.

Previous research has assessed suicide trends in Colombia and shown a shift between risk groups by gender and age group. In Colombia, from 1970 to 1990, the number of suicides reported among people under 20 years of age doubled, while the suicide rate exceeded several natural causes of death ^4^. The highest incidence of suicide from 1985 to 2002 was recorded among people aged 10 to 29 years, and this incidence showed a sustained increase from 1998 to 2002 ^5^. From 2000 to 2010, there was a decrease in suicide, with young adults (20-29) accounting for most cases and the Andean region reporting 60.8% of cases, evidencing the disparities between age groups and locations ^6^. Lemus Aponte ^7^ found the highest proportion of suicide in those aged 20 to 29 years, and around 30% of cases occurred in Antioquia and Bogotá from 2004 to 2018, with evidence of an increase since 2014. Previous research reported that male adults over 29 years of age were at the highest risk of suicide in rural areas from 2016 to 2017 ^8^. Murillo Gutiérrez et al. ^9^ assessed risk factors for suicide attempts from 2016 to 2019 and showed an increase in incidence mainly in females in capital cities. 

The COVID-19 pandemic changed the dynamics of several health problems, and suicide was no exception. A previous online survey showed a perception of higher suicidal ideation during the COVID-19 lockdown, but without considering the total number of cases in the country ^10^. Nevertheless, Peña et al. ^11^ found that the suicide mortality rate decreased in 2020, despite previous trends showing an increase in suicide cases since 2008. Franco-Ramírez et al. ^12^ also found a decrease in the suicide rate during the COVID-19 lockdown in the Eje Cafetero region, which had a higher incidence of suicide than other regions.

COVID-19 also exposed people to depression and anxiety, and the mass media contributes to the publication of descriptions of suicide practices, which may encourage imitation. Palacios-Espinosa et al. ^13^ evaluated the mass media during and before COVID-19 in Colombia and found that the media can induce suicidal behavior and also contribute to education on and prevention of these episodes. A survey in 2020 found that COVID-19 news had an association with suicidal ideation, while spiritual content could help prevent suicide, illustrating the effect of the media on suicide ^14^.

Although COVID-19 affected the previous trends of suicide, the effects per age group and region had taken divergences before the lockdown, and the analysis of spatial and temporal patterns per age group can help design control programs. For this reason, we implemented spatiotemporal models to illustrate the risk pattern of suicide attempts in Colombia before and during the COVID-19 in 2020 divided by age groups. We also included the effect of internet coverage as a predictor of media on suicide in Colombia.

## Materials and methods

A descriptive analysis of suicide attempts in Colombia from 2018 to 2020 was conducted, using a Bayesian model to represent the spatiotemporal risk by age and the effect of internet coverage.

### Data

A database of 85,526 individual records of suicide attempts from 2018 to 2020 was used, and each record contained information on date of notification, municipality (1,121 municipalities), gender, and age. The Colombian National Health Institute (INS, acronym in Spanish) provided this database responding to public information ^15^. The records were divided into six age groups: 5-9 years, 10-14 years, 15-19 years, 20-24 years, 25-59 years, and > 59 years. The Colombian National Administrative Department of Statistics (DANE, acronym in Spanish) provided the estimated population per municipality based on the *National Census* of 2018, the geographical division, and the municipal codes of Colombia via datasets and maps ^16^. The census of 2018 was selected because it included the population with the corresponding age, which made it possible to calculate the division into the six age groups of suicide attempts.

Another tool used in the study was a database with the number of fixed internet points per inhabitant in the 1,121 municipalities of Colombia per trimester from 2018 to 2020. This database was provided by the Colombian Ministry of Information and Communication Technologies (MinTIC, acronym in Spanish) via the open data website of the Colombian government ^17^.

### Descriptive analysis

Descriptive figures such as time series and maps were used to describe suicide attempts in cases over time and incidence rates per year. These figures were implemented in R (http://www.r-project.org) using the *tidyverse* and *rgdal* packages ^18^ ^,^ ^19^.

### Bayesian models

Three Bayesian models were used to represent suicide attempts by age group: Poisson; zero-inflated Poisson; and negative binomial. These models were implemented using the integrated nested Laplace approximation (INLA), which provides computational efficiency for running Bayesian models ^20^ ^,^ ^21^. The models with the lowest deviance information criterion (DIC) per age group were selected. This criterion evaluates the quality of the Bayesian model fit.

(a) Poisson model: a Bayesian model was implemented to represent suicide attempts in municipality *i* in quarter *t* (*y*_*it*_ *)*. *i* denotes the spatial division of municipalities in Colombia (*i* = *{*1,..., 1121*}*) and *t* denotes 12 quarters from the first quarter of 2018 to the fourth quarter of 2020 (*t* = *{*1,..., 12*}*). *y*_*it*_ was modeled as counts using a Poisson distribution with mean *λ*_*it*_ .

$$y_{it}∼Poisson\left(\lambda_{it}\right)$$ (1)

$$\lambda_{it}=\rho_{it}\epsilon_{it}$$,

in which *ρ*_*it*_ is the incidence rate of suicide attempts and

$$\epsilon_{it}$$

is an offset defined as the population per age group in Colombia in municipality *i* in quarter *t*. Was modeled *ρ*_*it*_ as a linear predictor in a logarithmic scale:

$$\nu_{it}=log\left(\rho_{it}\right)=\alpha +\gamma_{i}+\delta_{t}+\beta_{i}\times I_{it}$$ (2)

in which α is the average incidence of suicide attempts in all municipalities, *γ*_*it*_ is the spatial-random effect according to independent Gaussian random effects (IID), *δ*_*t*_ is the temporal-random effect according to the Bernardinelli et al. ^22^ characterization using the Random Walk Model of order two (rw2) ^23^, *β*_*i*_ is the random effect of the increase in fixed wireless internet points over the rate of suicide attempts rate in municipality *i*, and *I*_*it*_ is the number of fixed wireless internet points per inhabitant in municipality *i* in quarter *t*. *γ*_*i*_ was analyzed to estimate the suicide attempt risk in all municipalities, *δ*_*t*_ was analyzed to evaluate the temporal evolution of suicide attempts, and *β*_*i*_ was assessed to estimate the effect of the installation of fixed wireless internet points on the incidence of suicide attempts. Independence was assumed for suicide attempts among municipalities.

(b) Zero-inflated Poisson model: *γ*_*it*_ also represents the suicide attempts in municipality *i* in quarter *t* as the counts in the Poisson model joining the mass distribution at zero (*ζ*_*0*_ ):

$$\begin{array}{cc} y_{it}∼Poisson\left(\lambda_{it}\right), & ifU_{it}>0\\ ∼\zeta_{0} & ifU_{it}=0 \end{array}$$ (3)

in which *U*_*it*_ is the indicator of the excess of zeros in municipality *i* in quarter *t* and *λ*_*it*_ is the mean of the Poisson distribution as in the previous model. This model also implements the linear predictor *v*_*it*_ as the Poisson model presents in Equation 2.

(c) Negative binomial model: *y*_*it*_ also represents the suicide attempts in municipality *i* in quarter *t* as the counts in previous models, and the negative binomial distribution considers a second parameter for involving the overdispersions in a Poisson model. This distribution has the mean *λ*_*it*_ scale parameter *ϕ*, and variance function

$$V\left(\lambda_{it}\right)=\lambda_{it}+\lambda_{it}^{2}/\phi$$:

$$y_{it}∼NegBin\left(\lambda_{it},\phi \right)\alpha_{it}>0,\phi >0$$ (4)

$$log\left(\lambda_{it}\right)=log\left(\epsilon_{it}\right)+\alpha +\gamma_{i}+\delta_{t}+\beta_{i}\times I_{it}$$ (5)

in which ϵ 𝑖𝑡 is the offset based on the population per age group per municipality and α, y_*i*_ , δ_*i*_ , β_*i*_ , and I_*it*_ capture the same random effects as the Poisson model.

## Results

**Figure 1:**
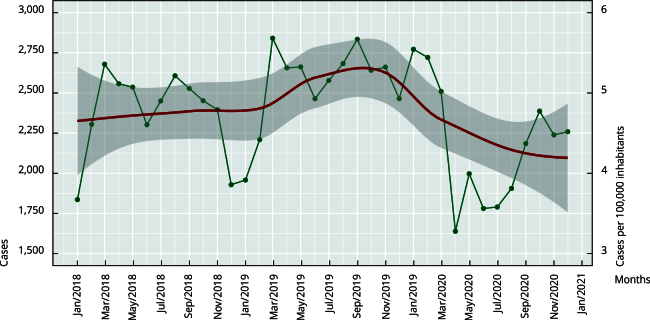
Monthly cases of suicide attempts and incidence per 100,000 inhabitants in Colombia from 2018 to 2020.

Note: the green line represents the occurrence of suicide attempts and the red line represents the trend in suicide attempts.

Colombia showed an increase in the incidence of suicide attempts from 2018 to 2019. This incidence then decreased in the first semester of 2020, as shown in Figure 1. Although April, June, and July 2020 had the slightest incidence of all time periods, the subsequent months of 2020 recovered the previous trends. Nevertheless, suicide attempts maintained a similar incidence, with 2,373 monthly cases on average.

**Figure 2:**
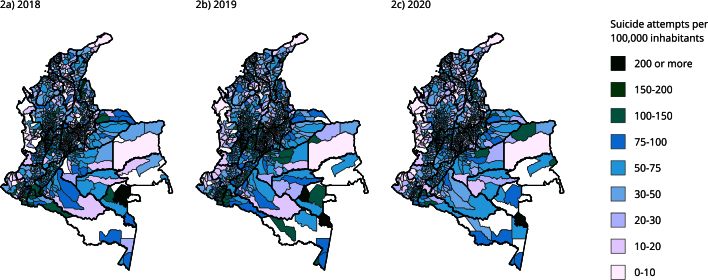
Annual incidence of suicide attempts per 100,000 inhabitants in Colombia in 2018, 2019, and 2020.

The incidence of suicide attempts per municipality in Colombia was in the central, southwestern, and southeastern regions, as shown in Figure 2. The central region had an incidence of more than 100 cases per 100,000 inhabitants, and this region corresponds to the territories of northern Tolima, Quindío, Risaralda, the central and southern regions of Antioquia, and the northwestern region of Cundinamarca and Boyacá. Although this region had more municipalities with an incidence of more than 100 cases per 100,000 inhabitants in 2019, the incidence decreased in 2020. The southwestern region of Colombia also showed an incidence of more than 100 cases per 100,000 inhabitants between the territories of southern Cauca, eastern Nariño, and Putumayo. The southeastern region had the highest incidence, with over 200 cases per 100,000 inhabitants in Vaupés. The northern (Caribbean region), western (Pacific region), and southeastern regions of Colombia had the municipalities with the lowest incidence, and the country as a whole had the lowest incidence in 2020.

### Bayesian models

**Figure 3:**
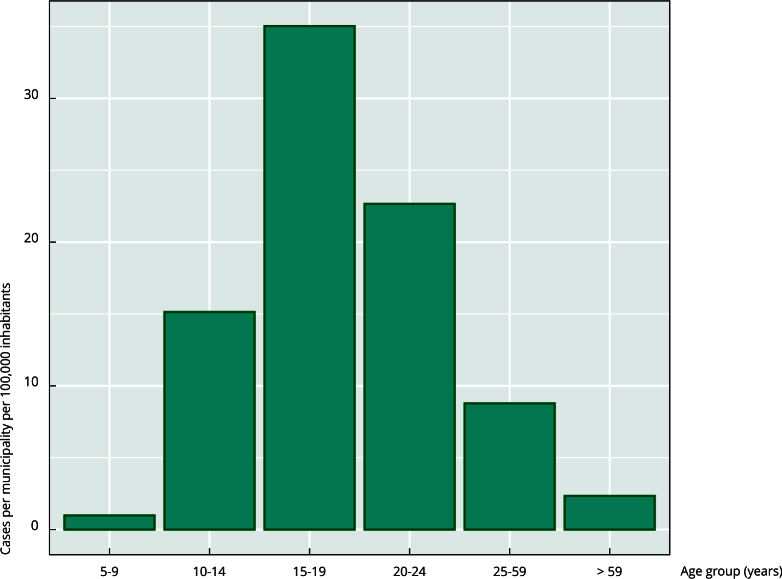
Average incidence of suicide attempts per 100,000 inhabitants per age group per trimester.

We ran three models per age group, selecting the model with the lowest DIC per group according to Table 1. The fixed effect of the models showed the highest incidence in overall municipalities per trimester in the population aged 15 to 19 years, as shown in Figure 3. This implies that all Colombian municipalities maintained a fixed incidence of suicide attempts of around 35 cases per 100,000 inhabitants in this age group, and the population groups aged 10 to 14 years and 20 to 24 years maintained incidences of 15 to 22 cases per 100,000 inhabitants per group per trimester. The population groups aged 5-9 years and > 59 years had the lowest fixed effect, with an incidence of more than 5 cases per 100,000 inhabitants. The group aged 25-59 years had a fixed incidence of 8.77 cases per 100,000 inhabitants, which shows that suicidal behavior had a lower impact in adults than in adolescents and young adults.

**Table 1:** Model criterion per age group.

Age group (years)/Model	WAIC	DIC
5-9		
Poisson	3,186.158	3,195.277
Negative binomial	3,187.307	3,97.524
Zero-inflated Poisson	4,312.337	4,311.514
10-14		
Poisson	18,230.26	18,186.16
Negative binomial	18,090.34	18,110.88
Zero-inflated Poisson	22,621.62	22,559.58
15-19		
Poisson	27,035.09	26,818.57
Negative binomial	26,774.53	26,775.47
Zero-inflated Poisson	32,527.08	32,310.89
20-24		
Poisson	20,305.29	20,239.65
Negative binomial	20,160.31	20,201.11
Zero-inflated Poisson	25,594.12	25,497
25-59		
Poisson	28,367.97	28,105.71
Negative binomial	28,115.6	28,128.04
Zero-inflated Poisson	34,073.34	33,813.42
> 59		
Poisson	6,926.692	6,929.841
Negative binomial	6,902.892	6,916.84
Zero-inflated Poisson	9,348.533	9,338.185

DIC: deviance information criterion; WAIC: Watanabe-Akaike information criterion.

**Figure 4:**
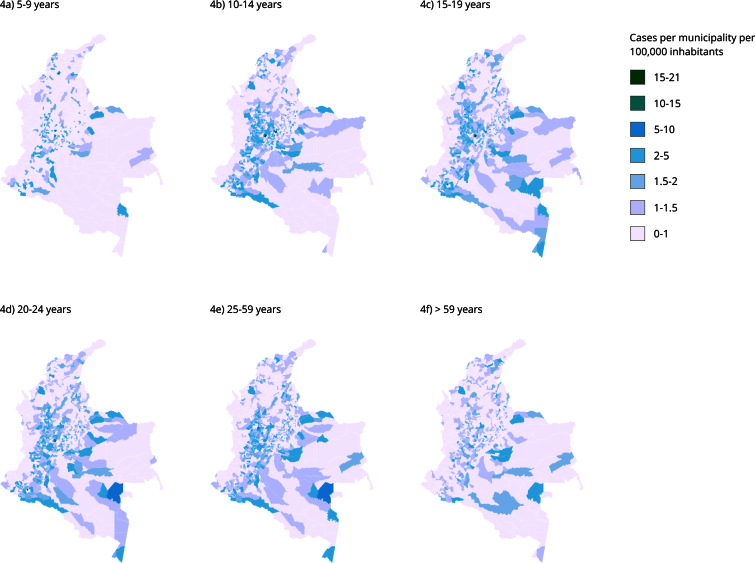
Incidence of suicide attempts per 100,000 inhabitants according to the spatial random effect.

The spatial random effect per model identified municipalities with incidences of over 2 cases per 100,000 inhabitants in the population group aged 10 to 59 years, mainly in the Andean region of Colombia (Figure 4). The Eje Cafetero region of Colombia, in the states of Antioquia, Risaralda, Caldas, Quindío, the east of Valle del Cauca and the west of Cundinamarca, and Putumayo had more municipalities with these conditions for the population group aged 10-14 years compared with the older population groups. Colón (Putumayo), Pacho (Cundinamarca), and Lérida (Tolima) had the highest incidence (more than 10 cases per 100,000 inhabitants) for the groups aged 10-24 years.

Facatativá (Cundinamarca), Santa Bárbara (Magdalena), La Virginia (Risaralda), Lérida, and Mariquita (Tolima) had the highest incidence for the population aged 5-9 years, with 5 to 10 cases per 100,000 inhabitants. La Virginia also maintained this incidence for all age groups except the last. El Rosal (Cundinamarca), Cáqueza (Cundinamarca), Albán (Nariño), Filandia (Quindío), and La Virginia also had this incidence for the group aged 10-14 years. San José (Antioquia), Pacho, and La Virginia also showed this incidence for the population aged 15-19 years. Two municipalities had this incidence for the group aged 20-24 years (Mitú-Vuapés, La Virginia), and five observed this incidence for the population aged 25-59 years (Abejoral-Antioquia, Colón, La Virginia, Lérida, Mitú).

We calculated the posterior probability of suicide attempts using the spatial random effect and identified 71, 298, 348, 306, 357, and 114 municipalities with posterior probabilities above 0.8 (high risk) per age group, respectively (Table 2 and Supplementary Material - Box S1. https://cadernos.ensp.fiocruz.br/static//arquivo/supl-e00119323_1694.pdf). Although Cundinamarca, Atlántico, Valle del Cauca, and Antioquia had the most municipalities at risk in the population group aged 5 to 9 years, Arauca, Atlántico, Quindío, Risaralda, and Vaupés had a higher proportion of municipalities at risk compared with the total. Antioquia, Arauca, Atlántico, Caldas, Cundinamarca, Huila, Meta, Nariño, Putumayo, Armenia, Risaralda, Tolima, and Valle del Cauca had more than 30% of municipalities at risk in the group aged 10-14 years, with Quindío and Caldas showing the highest proportion. Antioquia, Boyacá, and Cundinamarca had the highest number of municipalities at risk in the group aged 15-19 years, and Arauca, Caldas, Huila, Putumayo, Quindío, Risaralda, Valle del Cauca, and Vaupés had more than 50% of municipalities at risk. The same states had more than 50% of municipalities at risk as the previous group, except Huila for the group aged 20-24 years. Although the spatial effect of the group aged 25 to 59 years had lower incidences than the groups aged 10-24 years, this age group had higher proportions of municipalities at risk than the other groups, and Antioquia, Cundinamarca, Boyacá, Huila, Tolima, and Valle del Cauca had most municipalities at risk. Lastly, the older age group evidenced 114 municipalities at risk, mainly in Antioquia, Cundinamarca, Huila, Tolima, and Valle del Cauca; and Arauca, Guaviare, Huila, Quindío, and Risaralda had more than 50% of municipalities at risk for this age group.

**Table 2:** Number of municipalities with posterior probability above 0.8.

State	Number of municipalities per age group (years)						
	Total	5-9	10-14	15-19	20-24	25-59	> 59
Amazonas	11	0	0	3	2	2	0
Antioquia	125	7	45	56	50	51	12
Arauca	7	3	3	4	5	5	3
Archipiélago de San Andrés, Providencia y Santa Catalina	2	0	0	0	0	0	0
Atlántico	23	6	7	9	6	10	3
Bogotá DC	1	0	0	0	0	0	0
Bolívar	46	1	8	5	7	4	1
Boyacá	123	2	14	27	15	24	5
Caldas	27	2	18	19	18	18	3
Caquetá	16	1	1	1	2	1	2
Casanare	19	1	4	2	4	3	1
Cauca	42	1	7	10	8	10	2
Cesar	25	2	3	6	7	6	3
Chocó	30	0	0	2	1	2	1
Córdoba	30	2	2	7	4	4	3
Cundinamarca	116	11	46	36	29	36	14
Guainía	8	0	0	1	0	1	0
Guaviare	4	0	1	2	1	2	1
Huila	37	2	17	20	17	26	8
La Guajira	15	0	1	2	3	3	1
Magdalena	30	1	5	2	3	2	3
Meta	29	3	9	11	13	9	4
Nariño	64	3	22	24	19	18	5
Norte de Santander	40	1	3	5	8	6	5
Putumayo	13	1	7	8	9	9	2
Quindío	12	4	11	10	7	9	4
Risaralda	14	3	7	13	10	14	4
Santander	87	3	10	9	6	19	5
Sucre	26	1	6	5	2	6	3
Tolima	47	4	20	23	23	29	9
Valle del Cauca	42	5	21	22	25	24	6
Vaupés	6	1	0	3	2	3	1
Vichada	4	0	0	1	0	1	0
Total	1,121	71	298	348	306	357	114

**Figure 5:**
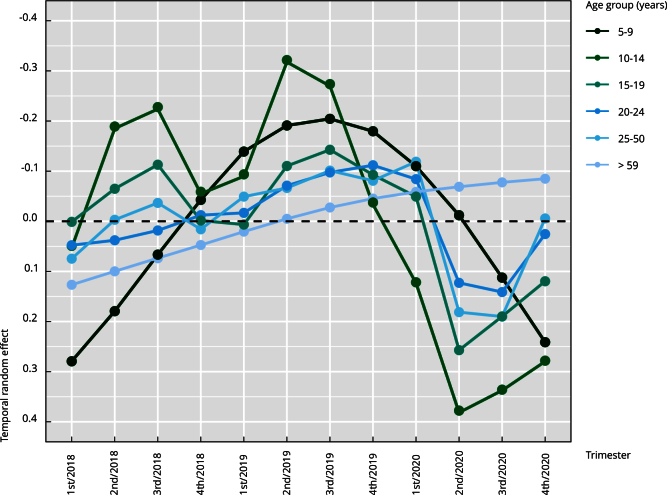
Temporal random effects per age group, Colombia.

Although the temporal random effect did not have the same impact as the fixed and spatial random effects, it did show a decrease in suicide trends from the fourth trimester of 2019, except in the group aged > 59 years (Figure 5). This result suggests that the COVID-19 outbreak may have reduced the occurrence of suicide attempts at a low level. The group aged 10-19 years showed a seasonal trend, with a higher incidence in the second and third trimesters and a lower incidence in the first and last trimesters. The group aged 5-9 years experienced the highest increase in the temporal effect until 2019, and the groups aged 20-59 years showed a similar trend. The oldest age group experienced an increase throughout the study period. Although this random effect evidenced a temporal dynamic between age groups, its impact was lower than 1.34 cases per 100,000 inhabitants in all groups.

We did not find a general relationship between fixed wireless internet points and the incidence of suicide attempts in Colombia, because the effect of this variable did not have positive or negative 0.025 and 0.975 quantiles in any municipality in each age group model.

## Discussion

This study proposed a spatiotemporal analysis of suicide attempts in Colombia from 2018 to 2020, aiming to identify suicide attempt patterns by municipality and region. The central, southwestern, and southeastern areas of Tolima, Quindío, Risaralda, and central and southern Antioquia showed a high incidence of suicide attempts, with over 100 cases per 100,000 inhabitants, as well as the northwestern region of Cundinamarca and Boyacá. Although several municipalities in the central region reported incidences of suicide attempts above the threshold in 2019, our study reveals that the overall incidence decreased in these areas in 2020. This finding suggests that there was a behavioral change in factors that contribute to suicide attempts in these areas during the study period. Previous work has also shown higher suicide attempt rates in the central, southwestern, and southeastern regions, in line with the current findings ^24^. Previous studies found that Risaralda had the highest suicide rates, and our study also found a high incidence of suicide attempts in this state ^25^. Previous work has highlighted a sustained decrease in suicide rates in Colombia since 2000, while current work shows an increase in incidence before 2020 ^26^. In the years following 2020, the Colombian government warned of an increase in suicides, evidencing the need to develop studies on suicide trends ^27^.

Colombia experienced an increase in suicide attempt rates from 2018 to 2019, followed by a decline in the first half of 2020. Although April, June, and July 2020 had the lowest incidence of suicide attempts of all time periods, the subsequent months of 2020 experienced a resurgence of previous trends. Nevertheless, the incidence of suicide attempts remained constant, averaging 2,373 cases per month. Chen et al. ^28^ also observed a decrease in overall suicide rates in Taiwan following the COVID-19 outbreak, which is consistent with the findings of this study. Similarly, South Korea reported an increase in suicide risk at the onset of the pandemic from February to December 2020, followed by a decrease in suicide rates in subsequent years ^29^. Data from Seoul indicate a decrease in depression severity and suicidal tendencies in the population in 2021 compared with 2020 ^30^.

Our results reveal variations in the incidence of suicide attempts in different regions of Colombia during COVID-19. The southwestern region had an incidence of more than 100 cases per 100,000 inhabitants, and the southeastern region showed the highest incidence, with over 200 cases per 100,000 inhabitants in Vaupés. On the other hand, the northern, western, and southeastern regions of Colombia had municipalities with the lowest incidence, contributing to the country’s overall lowest incidence in 2020. In relation to suicidal behavior, previous work has reported high rates of suicidal ideation in Bogota, with mental illness and displacement by violence identified as contributing factors ^31^. We found that the southwestern region of Colombia had the highest rates, which is in line with the results of previous studies ^32^. Previous work showed that some regions of Brazil had the highest incidence of suicide among older adults in 2020, which is consistent with our findings of an unaltered upward trend in suicide attempts among people aged > 59 years ^33^. The Caribbean region, excluding Guyana and Trinidad and Tobago, reported relatively low suicide rates, as was the case in the Western Pacific and Caribbean regions of Colombia, where we found the lowest suicide rate among individuals aged over 59 years ^34^. These findings suggest that the Caribbean region, particularly the Western Pacific, may have a lower incidence of both COVID-19 and suicidal behavior than other locations ^35^. The temporal random effect highlighted a decrease in suicide trends starting from the first quarter of 2020, except in the population aged > 59 years.

It was found that individuals aged 15-19 years were at the highest risk of attempting suicide, with a consistent quarterly incidence of approximately 35 cases per 100,000 inhabitants across all municipalities. This finding is in line with the work of Christoffel et al. ^36^, who found the highest incidence of suicide attempts in this age group, especially towards the end of the school year. The population groups aged 10-14 years and 20-24 years also had higher incidences, with 15 to 22 cases per 100,000 inhabitants per quarter in all municipalities, and Colombia has maintained this higher risk group in 2023 ^37^. In contrast, the groups aged 5-9 years and those aged > 59 years had the lowest incidence (5 cases per 100,000 inhabitants). This finding suggests that suicide attempts vary among age groups in Colombia. These results show a high risk among adolescents in Colombia, which has also been observed in regions such as Asia, in contrast to Europe, where the highest risk of suicide was found among older adults ^35^. Our results contrast with the global suicide incidence, which showed the highest mortality rate in individuals aged 0-15 years, suggesting the disparity of the Colombian population compared with global trends in 2019 ^38^.

The spatial random effects model identified municipalities with an incidence of more than 2 cases per 100,000 inhabitants aged 10-59 years, mainly in the Andean region of Colombia (Figure 4). The Eje Cafetero region of Colombia (Antioquia, Risaralda, Caldas, Quindío, the eastern part of Valle del Cauca, and the western part of Cundinamarca), as well as Putumayo, showed more municipalities with these conditions in the group aged 10-14 years compared with subsequent age groups, in line with the work of Quemba Mesa et al. ^39^, who found an increase in suicide attempt rates in children and adolescents aged 6-17 years from 2016 to 2019. This study also provides a set of risk factors in this age group, such as being female, living in the city center, being ethnic group, being a migrant, being a victim of violence, being displaced, and being in government programs. Previous work has shown that Huila and Quindío had a age-standardized suicide mortality rate that was 1.71 times higher than the national average from 2010 to 2013, and the spatial risk showed several municipalities with a higher risk for people aged 10-59 years ^25^.

Our results also showed that some municipalities obtained the highest incidence of suicide attempts in several age groups of young adults, which demonstrates the need for health efforts in these regions. Mitú and La Virginia showed an incidence of 5 to 10 cases per 100,000 inhabitants in the group aged 20-24 years due to the spatial effect, and five municipalities showed a similar incidence in individuals aged 25-59 years (Abejoral-Antioquia, Colón, La Virginia, Lérida, Mitú). A large proportion of the population of Mitú is Indigenous, and previous work has highlighted this ethnic condition as a risk factor for suicide due to the sociocultural practices of this population ^40^. Medina-Pérez & Escobar ^41^ characterized the epidemiology of suicide among young adults in the department of Risaralda and found a high prevalence among men, a significant proportion of cases in Pereira, and an impact of environmental factors on suicide attempts among young adults in Colombia ^41^. La Virginia is a municipality in the state of Risaralda, and previous work only found the highest incidence of suicide in this place in 2010, suggesting that suicidal behavior increased in La Virginia in subsequent years ^25^. The municipality of Colón, in the state of Putumayo, has maintained a high incidence of suicide, as shown by previous work with data from 1998 to 2017, and this state also has a higher proportion of Indigenous people ^42^. Lerida found a higher risk of suicide in the state of Tolima, and previous work has analyzed data from a health center that indicate that being a woman and taking toxic substances are the most common features in reports of suicide attempts ^43^.

Our results highlight the need to consider age-specific prevention strategies according to specific risk factors across the life course. Woods et al. ^44^ highlighted the association between suicide attempts and other health risk behaviors, including depression, substance use, and impulsive behavior in adolescents. On the other hand, Arenas et al. ^31^ found that mental illness, displacement due to violence, and substance use increased suicidal behavior in Colombian adults. Previous work has examined the relationship between substance use and suicide risk in young adults and identified cannabis and tobacco use as risk factors ^45^. Substance use also affects countries such as Mexico, where a previous study highlighted the role of traumatic life events, school disengagement, and tobacco use in the prediction of suicidal behavior during the transition from adolescence to adulthood ^46^. These findings support the notion that addressing risk factors is criticial in defining suicide prevention efforts.

We did not find a general relationship between fixed points of wireless internet and the incidence of suicide attempts in Colombia, because the effect of this variable lacks both positive and negative quantiles at 0.025 and 0.975 in any municipality in each age group model. A longitudinal study had already reported that suicide-related behavior on the internet did not predict suicidal ideation ^47^. However, Sakarya et al. ^48^ found that some websites have pro-suicidal properties. Although our study evaluated the effect of the internet on suicide, this model could evaluate other causes using the same approach. Our study limited the media diffusion pathway to the fixed internet points, but smartphones also play a role. Shinetsetseg et al. ^49^ found a higher risk of suicidal ideation and attempts among adolescents with smartphone addiction in Republic of Korea.

This work has a number of limitations related to COVID-19 disturbances, data quality, and lack of sociodemographic data analysis. First, the temporal scope of the research included the isolation period of the pandemic, which interfered with the suicide attempt reports and the record of previous trends in suicide attempt rates. The accuracy of reported data is another limitation, as differences in reporting practices and potential underreporting in different regions could affect the results. In addition, future research should assess the specific influences of pandemic-related factors on suicidal behavior. Regional heterogeneity is another limitation, as the study identified patterns without fully elucidating the diverse cultural, socioeconomic, and local circumstances that may contribute to variations in suicide attempt rates across regions. Although the age group analysis provides insight into specific cohorts, subsequent work should highlight the causes contributing to the trends observed within each age group. These limitations underscore the need for caution in generalizing our findings and highlight avenues for further research to improve the depth and specificity of our understanding of suicide attempt patterns in Colombia.

The results of this study highlight the dynamics of suicide attempts in Colombia, revealing temporal, spatial, and age variations. While certain months in 2020 showed a temporary decrease in the incidence of suicide, the subsequent months witnessed a resumption of previous trends. Our results are consistent with international studies, such as those in Taiwan and Republic of Korea, which suggest a complex interplay between the COVID-19 pandemic and suicide rates. Geographically, our study identified specific regions in Colombia where the incidence of suicide attempts exceeded 100 cases per 100,000 inhabitants, specifically the central, southwestern, and southeastern areas. Interestingly, despite the higher incidence reported in these regions in 2019, our study found a decrease in overall incidence in 2020, indicating a potential positive shift in contributing factors. These regional patterns are consistent with existing literature on suicide attempts in Colombia, emphasizing the importance of considering geographical variations in prevention strategies. Lastly, our study provides age-specific insight, highlighting a concentration of suicide attempts among individuals aged 15-19 years. These results align with previous research and emphasize the need for targeted interventions in this vulnerable age group. The nuanced breakdown of incidence across age groups further underscores the importance of tailoring prevention strategies based on age-specific risk factors.

## References

[1] World Health Organization (2021). Suicide worldwide in 2019: global health estimates.

[2] Ministerio de Salud y Protección Social (2018). Boletín de salud mental: conducta suicida.

[3] Ministerio de Salud y Protección Social (2022). Salud mental: asunto de todos.

[4] Vásquez R, Gómez DL (1993). Mortalidad y problemas emocionales el suicidio en Colombia 20
años después (1970-1990). Acta Méd Colomb.

[5] Cendales R, Vanegas C, Fierro M, Córdoba R, Olarte A (2007). Tendencias del suicidio en Colombia, 1985-2002. Rev Panam Salud Pública.

[6] Cardona Arango D, Medina-Pérez OA, Cardona Duque DV (2016). Characterisation of suicide in Colombia,
2000-2010. Rev Colomb Psiquiatr.

[7] Lemus Aponte M (2020). Tendencias en los patrones de suicidio en Colombia: 2004 a 2018.

[8] Moreno LSC, Valencia LFF, García OEP, Lozada CMM (2023). Risk factors associated with suicide attempt as predictors of
suicide, Colombia, 2016-2017. Rev Colomb Psiquiatr.

[9] Murillo Gutiérrez LC, Quemba Mesa MP, Vargas Rodríguez LY, Florez Escobar IC, Contreras Briceño JI (2022). Epidemiological behavior of suicide attempt in Colombian
adolescents years 2016-2019: an ecological study.. Rev Latinoam Enferm.

[10] Caballero-Domínguez CC, Jiménez-Villamizar MP, Campo-Arias A (2022). Suicide risk during the lockdown due to coronavirus disease
(COVID-19) in Colombia. Death Stud.

[11] Peña JRZ, Peña JPZ, Landaeta APV, Ferro E, Marín AL, Bejarano DRC (2023). Temporal changes in suicide mortality rates before and during the
COVID-19 pandemic in Colombia. A joint point regression
model.. Rev Colomb Psiquiatr.

[12] Franco-Ramírez JD, Agudelo-Mejía K, Medina-Osorio JC, Moreno-Gómez G, Franco-Londoño J (2023). Impact of the lockdown by the COVID-19 pandemic on suicidal trend
in the Colombian Coffee Region. Heliyon.

[13] Palacios-Espinosa X, Hernández DAL, Gutiérrez FM (2024). Coverage of suicide in traditional media in colombia, before and
during the pandemic (2018-2021). Revista Latina de Comunicación Social.

[14] Garcés-Prettel ME, Barredo-Ibañez D, Arroyave-Cabrera J, Santoya-Montes Y (2023). Suicide risk and media consumption in the COVID-19 pandemic in
Colombia. Revista de Comunicación.

[15] Instituto Nacional de Salud Sistema de Vigilancia en Salud Pública - SIVIGILA..

[16] Departamento Administrativo Nacional de Estadística Geoportal DANE..

[17] Ministerio de Tecnologías de la Información y
Comunicaciones Internet fijo penetración municipio..

[18] Wickham H, Averick M, Bryan J, Chang W, McGowan LD'A, François R (2019). Welcome to the Tidyverse.. J Open Source Softw.

[19] Keitt T, Bivand R, Pebesma E, Rowlingson B rgdal: bindings for the Geospatial Data Abstraction Library..

[20] Blangiardo M, Cameletti M, Baio G, Rue H (2013). Spatial and spatio-temporal models with R-INLA. Spat Spatiotemporal Epidemiol.

[21] Rue H, Martino S, Chopin N (2009). Approximate Bayesian inference for latent Gaussian models by
using integrated nested Laplace approximations. J R Stat Soc Ser B Stat Methodol.

[22] Bernardinelli L, Clayton D, Pascutto C, Montomoli C, Ghislandi M, Songini M (1995). Bayesian analysis of space-time variation in disease
risk. Stat Med.

[23] Lindgren F, Rue H (2008). On the second-order random walk model for irregular
locations. Scand J Stat.

[24] Blandón Rodríguez AM, Chaves Torres NM (2020). High prevalence of two or more suicide attempts associated with
suicidal ideation and mental disease in Colombia 2016. Rev Colomb Psiquiatr.

[25] Rodríguez-Escobar JA, Medina-Pérez OA, Cardona-Duque DV (2013). Caracterización del suicidio en el departamento de Risaralda,
Colombia, 2005-2010. Revista de la Facultad de Medicina.

[26] Chaparro-Narváez P, Díaz-Jiménez D, Castañeda-Orjuela C (2019). The trend in mortality due to suicide in urban and rural areas of
Colombia, 1979-2014. Biomedica.

[27] Procuraduría General da la Nación (2023). Suicidio disparado en Colombia por cuenta de trastornos
mentales.

[28] Chen YY, Yang CT, Pinkney E, Yip PSF (2022). Suicide trends varied by age-subgroups during the COVID-19
pandemic in 2020 in Taiwan. J Formos Med Assoc.

[29] Min J, Oh J, Kim SI, Kang C, Ha E, Kim H (2022). Excess suicide attributable to the COVID-19 pandemic and social
disparities in South Korea. Sci Rep.

[30] Lee J, Ko YH, Shin C, Han R, Chae N, Yoon HK (2022). Suicide and suicide prevention awareness in Korea during the
COVID-19 pandemic. Psychiatry Investig.

[31] Arenas A, Gómez-Restrepo C, Rondón M (2016). Factores asociados a la conducta suicida en Colombia Resultados
de la Encuesta Nacional de Salud Mental 2015. Rev Colomb Psiquiatr.

[32] Silbato MPP, Gómez YO, Martínez MIG (2009). El suicidio en Nariño una mirada desde los observatorios del
delito en cinco municipios del Departamento. Pensamiento Psicológico.

[33] Pinto LW, Assis SG, Pires TO (2012). Mortalidade por suicídio em pessoas com 60 anos ou mais nos
municípios brasileiros no período de 1996 a 2007. Ciênc Saúde Colet.

[34] Emmanuel MK, Campbell MH (2012). Commentary homicide-suicide in the Caribbean. J Am Acad Psychiatry Law.

[35] Värnik P (2012). Suicide in the world. Int J Environ Res Public Health.

[36] Christoffel KK, Marcus D, Sagerman S, Bennett S (1988). Adolescent suicide and suicide attempts a population
study. Pediatr Emerg Care.

[37] Departamento Nacional de Planeación; Consejería Presidencial para la
Equidad de la Mujer; Observatorio Colombiano de las Mujeres (2023). El suicidio en Colombia: factores diferenciales entre mujeres y
hombres..

[38] World Health Organization (2023). World health statistics 2023: monitoring health for the SDGs,
Sustainable Development Goals.

[39] Quemba Mesa MP, Herrera Tarapues JC, Mendoza Ortiz A, Mendoza Ortiz B (2022). Comportamiento epidemiológico del intento de suicidio en niños y
adolescentes, Colombia 2016-2020. Pediatría.

[40] Martínez Sïlva PA, Dallos Arenales MI, Prada AM, Rodríguez Van der Hammen MC, Mendoza Galvis N (2020). Un modelo explicativo de la conducta suicida de los pueblos
indígenas del departamento del Vaupés, Colombia.. Rev Colomb Psiquiatr.

[41] Medina-Pérez OA, Escobar JAR (2012). Caracterización del suicido en adultos jóvenes del área
metropolitana del departamento de Risaralda, Colombia,
2005-2011. Revista Médica Electrónica.

[42] Medina-Pérez OA, Blandón-Cuesta OM, Barrera-Carvajal V (2021). Caracterización de adolescentes fallecidos por
suicidio. Rev Cub Med Mil.

[43] Cali Baleta NM, Yara DC, Zabala Rodríguez YK (2017). Caracterización de los casos de intento suicidio que ingresaron al
Hospital Especializado Granja Integral de Lérida Tolima en los años 2014 a
2016.

[44] Woods ER, Lin YG, Middleman A, Beckford P, Chase L, DuRant RH (1997). The associations of suicide attempts in
adolescents. Pediatrics.

[45] Pereira-Morales A, Adan A, Forero D (2019). Network analysis of multiple risk factors for mental health in
young Colombian adults. J Ment Health.

[46] Benjet C, Menendez D, Albor Y, Borges G, Orozco R, Medina-Mora ME (2018). Adolescent predictors of incidence and persistence of
suicide-related outcomes in young adulthood a longitudinal study of Mexican
youth. Suicide Life Threat Behav.

[47] Sueki H (2013). The effect of suicide-related Internet use on users' mental
health. Crisis.

[48] Sakarya D, Günes C, Sakarya A (2013). Googling suicide evaluation of websites according to the content
associated with suicide. Turk Psikiyatri Dergisi.

[49] Shinetsetseg O, Jung YH, Park YS, Park E-C, Jang S-Y (2022). Association between smartphone addiction and
suicide.. Int J Environ Res Public Health.

