# Supplementary material for: Spatiotemporal analysis of suicide attempts in Colombia from 2018 to 2020
Source: Cad Saude Publica. 2024 Sep 16;40(8):e00119323. doi: 10.1590/0102-311XEN119323 (PMC11405025; doi:10.1590/0102-311XEN119323)
Supplement: Supplementary file 1 [file 1678-4464-csp-40-08-EN119323-s.pdf]

Box S1 Municipalities with posterior probability above 0.8.

STATE	MUNICIPALITIES PER AGE GROUP (YEARS)					
	5-9	10-14	15-19	20-24	25-29	> 59
Amazonas	None	None	Puerto Nariño, Tarapacá, Leticia	Puerto Nariño, Leticia	Puerto Nariño, Leticia	None
Antioquia	Medellín, Don Matías, Retiro, Rionegro, Sabaneta, San Jerónimo, Támesis	Medellín, Abejorral, Andes, Anorí, Barbosa, Belmira, Bello, Betulia, Ciudad Bolívar, Cañasgordas, El Carmen De Viboral, Cocorná, Concordia, Envigado, Fredonia, Frontino, Girardota, Guarne, Guatapé, Itagüí, Ituango, Jardín, La Ceja, La Pintada, La Unión, Maceo, Rionegro, Sabaneta, Salgar, San Jerónimo, San José De La Montaña, San Luis, San Pedro De Los Milagros, Santa Rosa De Osos, Segovia, Sonsón, Támesis, Tarazá, Titiribí, Urrao, Valdivia, Valparaíso, Venecia, Yarumal, Yolombó	Medellín, Abejorral, Amalfi, Angostura, Anorí, Apartadó, Barbosa, Bello, Betania, Betulia, Ciudad Bolívar, Briceño, Campamento, Caramanta, El Carmen De Viboral, Cisneros, Cocorná, Concepción, Concordia, Don Matías, Ebéjico, Entrerríos, Envigado, Fredonia, Girardota, Gómez Plata, Guarne, Heliconia, Hispania, Itagüí, Ituango, Jardín, La Ceja, La Pintada, La Unión, Liborina, Marinilla, Nariño, Pueblorrico, Rionegro, Salgar, San Andrés De Cuerquía, San Jerónimo, San José De La Montaña, San Pedro De Los Milagros, San Rafael, Santa Rosa De Osos, El Santuario, Segovia, Sonsón, Támesis, Titiribí, Toledo, Urrao, Valdivia, Yarumal	Zaragoza, Medellín, Abejorral, Amalfi, Andes, Angostura, Apartadó, Belmira, Bello, Betulia, Ciudad Bolívar, El Carmen De Viboral, Carolina, Cisneros, Cocorná, Concordia, Don Matías, Envigado, Fredonia, Giraldo, Girardota, Gómez Plata, Guarne, Guatapé, Hispania, Itagüí, Ituango, Jardín, Jericó, La Ceja, La Unión, Liborina, Puerto Triunfo, Retiro, Rionegro, Salgar, San Jerónimo, San Pedro De Los Milagros, Santa Bárbara, Santa Rosa De Osos, El Santuario, Segovia, Sonsón, Támesis, Tarazá, Tarso, Toledo, Urrao, Valdivia, Yarumal	Medellín, Abejorral, Amagá, Amalfi, Andes, Angelópolis, Angostura, Anzá, Apartadó, Argelia, Barbosa, Belmira, Bello, Betulia, Ciudad Bolívar, El Carmen De Viboral, Carolina, Chigorodó, Cisneros, Concordia, Don Matías, Ebéjico, Envigado, Girardota, Granada, Guarne, Guatapé, Heliconia, Itagüí, La Ceja, La Pintada, Marinilla, Puerto Triunfo, Rionegro, Salgar, San Andrés De Cuerquía, San Jerónimo, San José De La Montaña, San Pedro De Los Milagros, Santa Rosa De Osos, El Santuario, Segovia, Sonsón, Sopetrán, Támesis, Titiribí, Urrao, Valdivia, Valparaíso, Vegachí, Yarumal	Medellín, Amalfi, Angostura, Barbosa, Don Matías, Envigado, La Ceja, Rionegro, El Santuario, Sonsón, Tarazá, Vegachí
Arauca	Arauca, Saravena, Tame	Arauca, Fortul, Tame	Arauca, Fortul, Saravena, Tame	Arauca, Puerto Rondón, Fortul, Saravena, Tame	Arauca, Puerto Rondón, Fortul, Saravena, Tame	Arauca, Fortul, Tame
Archipiélago De San Andrés,	None	None	None	None	None	None

Providencia y Santa Catalina						
Atlántico	Barranquilla, Baranoa, Galapa, Malambo, Repelón, Soledad	Barranquilla, Baranoa, Juan De Acosta, Ponedera, Sabanagrande, Sabanalarga, Soledad	Barranquilla, Baranoa, Galapa, Palmar De Varela, Ponedera, Puerto Colombia, Sabanalarga, Santa Lucía, Soledad	Barranquilla, Baranoa, Galapa, Puerto Colombia, Santa Lucía, Soledad	Barranquilla, Baranoa, Galapa, Juan De Acosta, Ponedera, Puerto Colombia, Sabanalarga, Santo Tomás, Soledad, Usiacurí	Barranquilla, Malambo, Puerto Colombia
Bogotá DC	None	None	None	None	None	None
Bolívar	San Jacinto	Cartagena De Indias, Arjona, Calamar, El Carmen De Bolívar, San Estanislao, Santa Rosa, Villanueva, Zambrano	Cartagena De Indias, Arjona, El Carmen De Bolívar, Santa Rosa, Santa Rosa Del Sur	Cartagena De Indias, Arjona, El Carmen De Bolívar, San Martín De Loba, Santa Catalina, Santa Rosa, Santa Rosa Del Sur	Cartagena De Indias, El Carmen De Bolívar, Santa Rosa, Santa Rosa Del Sur	Cartagena De Indias
Boyacá	Tunja, Duitama	Tunja, San Eduardo, San José De Pare, Soatá, Sora, Tota, Chiquinquirá, Puerto Boyacá, Sogamoso, Duitama, Garagoa, Guateque, Villa De Leyva, Monguí	Tunja, Almeida, Aquitania, Berbeo, Buenavista, Ramiriquí, San Eduardo, Santana, Sativasur, Siachoque, Tasco, Tibaná, Güicán De La Sierra, Chiquinquirá, Chita, Chitaraque, Coper, Puerto Boyacá, Sogamoso, Ventaquemada, Duitama, Gachantivá, Garagoa, Guateque, Jericó, Villa De Leyva, Maripí	Tunja, Belén, Santa Rosa De Viterbo, Siachoque, Sora, Tasco, Tibaná, Chiquinquirá, Chitaraque, Puerto Boyacá, Sogamoso, Duitama, Garagoa, Guateque, Villa De Leyva	Tunja, Briceño, Paipa, Ramiriquí, Santana, Siachoque, Toca, Tota, Turmequé, Tuta, Güicán De La Sierra, Chiquinquirá, Chita, Cómbita, Puerto Boyacá, Socha, Sogamoso, Duitama, Garagoa, Guateque, Villa De Leyva, Macanal, Miraflores, Moniquirá	Oicatá, Chiquinquirá, Puerto Boyacá, Sogamoso, Duitama
Caldas	Manizales, Norcasia	Manizales, Aguadas, Neira, Marquetalia, Marmato, Manzanares, La Dorada, Filadelfia, Chinchiná, Belalcázar, Aranzazu, Anserma, Viterbo, Villamaría, Supía, Pensilvania, Palestina, Riosucio	Manizales, Aguadas, Neira, Marquetalia, Manzanares, La Dorada, Filadelfia, Chinchiná, Belalcázar, Aranzazu, Anserma, Viterbo, Supía, San José, Samaná, Salamina, Pensilvania, Pácora, Riosucio	Manizales, Neira, Marulanda, Marquetalia, Marmato, La Dorada, Filadelfia, Chinchiná, Belalcázar, Aranzazu, Anserma, Viterbo, Villamaría, Supía, San José, Samaná, Pácora, Riosucio	Manizales, Aguadas, Neira, Marmato, Manzanares, La Dorada, Filadelfia, Chinchiná, Belalcázar, Aranzazu, Anserma, Viterbo, Villamaría, Supía, Salamina, Risaralda, Pensilvania, Riosucio	Manizales, La Dorada, Chinchiná
Caquetá	Puerto Rico	Florencia	Florencia	Florencia, Cartagena	Florencia	Solano, Florencia

				Del Chairá		
Casanare	Yopal	Yopal, Aguazul, Pore, Villanueva	Yopal, Tauramena	Yopal, Aguazul, Maní, Orocué	Yopal, Maní, Paz De Ariporo	Yopal
Cauca	Popayán	Florencia, Morales, Rosas, Santander De Quilichao, Toribío, El Tambo, Popayán	Florencia, Miranda, Patía, Piamonte, Rosas, Santander De Quilichao, Suárez, Toribío, Popayán, Caldono	Florencia, Inzá, Patía, Santander De Quilichao, Silvia, Toribío, Popayán, Caldono	Corinto, Inzá, Patía, Puracé, Rosas, Santander De Quilichao, Suárez, Timbío, Toribío, Popayán	Santander De Quilichao, Popayán
Cesar	Chiriguaná, Aguachica	El Copey, Valledupar, Aguachica	Curumaní, Pelaya, San Alberto, San Diego, Valledupar, Aguachica	La Jagua De Ibirico, Pelaya, Tamalameque, Valledupar, Pueblo Bello, San Martín, Aguachica	Pailitas, San Alberto, Valledupar, Becerril, San Martín, Aguachica	Pailitas, Valledupar, Aguachica
Chocó	None	None	Quibdó, El Cantón Del San Pablo	El Carmen De Atrato	Quibdó, El Carmen De Atrato	El Carmen De Atrato
Córdoba	Momil, San Antero	Montería, Cereté	Montería, Cereté, Cotorra, Lorica, Planeta Rica, Pueblo Nuevo, San Antero	Montería, Chinú, Ciénaga De Oro, Lorica	Montería, Lorica, San Antero, Montelíbano	Montería, Ayapel, Montelíbano
Cundinamarca	Beltrán, Zipaquirá, Girardot, Chía, Cajicá, El Colegio, Facatativá, Madrid, Venecia, Pacho, Quetame	Anolaima, Arbeláez, Sasaima, Silvania, Suesca, Tibacuy, Tocancipá, Topaipí, Une, Útica, Vianí, Yacopí, Zipaquirá, Agua De Dios, Girardot, Soacha, Chía, El Rosal, Mosquera, Fusagasugá, San Juan De Rioseco, Cajicá, Cáqueza, Carmen De Carupa, Chaguaní, Chipaque, El Peñón, Facatativá, Guachetá, Guaduas, Guataquí, Guayabal De Síquima, Gutiérrez, La Calera, La Mesa, La Vega, Madrid, Manta, Venecia, Pacho, Puerto Salgar, Pulí, Quipile, Ricaurte, San Antonio	Anapoima, Sasaima, Silvania, Suesca, Tocaima, Topaipí, Villa De San Diego De Ubaté, Vianí, Yacopí, Zipaquirá, Agua De Dios, Girardot, Chía, El Rosal, Fusagasugá, San Juan De Rioseco, Caparrapí, Cáqueza, Choachí, El Peñón, Facatativá, Fómeque, Fosca, Guaduas, Guatavita, Guayabetal, La Mesa, La Palma, La Vega, Madrid, Nemocón, Venecia, Pacho, Puerto Salgar, Apulo, San Antonio Del Tequendama	Anapoima, Arbeláez, Silvania, Tabio, Tocaima, Villa De San Diego De Ubaté, Viotá, Zipaquirá, Girardot, Soacha, Chía, El Rosal, Fusagasugá, Caparrapí, Cáqueza, Choachí, Chocontá, El Colegio, Facatativá, Guaduas, La Mesa, La Vega, Madrid, Nemocón, Pacho, Puerto Salgar, Apulo, San Antonio Del Tequendama, San Bernardo	Anolaima, Sesquilé, Silvania, Simijaca, Tocaima, Ubaque, Villa De San Diego De Ubaté, Vergara, Vianí, Villeta, Viotá, Zipaquirá, Agua De Dios, Girardot, Soacha, Chía, El Rosal, Fusagasugá, Cáqueza, Choachí, Chocontá, El Colegio, Facatativá, Gachetá, Guachetá, Guaduas, La Mesa, La Palma, La Vega, Madrid, Nemocón, Venecia, Pacho, Puerto Salgar, San Antonio Del Tequendama, San Cayetano	Silvania, Tocaima, Vianí, Girardot, Soacha, Chía, Fusagasugá, Chocontá, Facatativá, La Mesa, La Vega, Medina, Pacho, San Antonio Del Tequendama

		Del Tequendama, San Cayetano				
Guainía	None	None	Inírida	None	Inírida	None
Guaviare	None	San José Del Guaviare	San José Del Guaviare, Miraflores	San José Del Guaviare	San José Del Guaviare, Miraflores	San José Del Guaviare
Huila	Neiva, La Plata	Neiva, Gigante, Guadalupe, Isnos, La Plata, Nátaga, Palestina, Pitalito, Rivera, Saladoblanco, San Agustín, Suaza, Tello, Teruel, Yaguará, Garzón, Acevedo	Neiva, Gigante, Guadalupe, Isnos, La Argentina, La Plata, Nátaga, Paicol, Palestina, Pitalito, Rivera, Saladoblanco, Santa María, Tello, Teruel, Yaguará, Garzón, Campoalegre, Algeciras, Acevedo	Neiva, Hobo, Íquira, Isnos, La Argentina, La Plata, Oporapa, Pitalito, Rivera, San Agustín, Tello, Teruel, Timaná, Garzón, Campoalegre, Aipe, Acevedo	Neiva, Gigante, Guadalupe, Hobo, Isnos, La Argentina, La Plata, Nátaga, Oporapa, Palermo, Palestina, Pitalito, Saladoblanco, San Agustín, Santa María, Suaza, Tello, Timaná, Yaguará, Garzón, Colombia, Campoalegre, Algeciras, Aipe, Agrado, Acevedo	Neiva, Gigante, Íquira, Isnos, La Plata, Pital, Pitalito, Garzón
La Guajira	None	San Juan Del Cesar	Villanueva, San Juan Del Cesar	El Molino, Riohacha, San Juan Del Cesar	Barrancas, Riohacha, San Juan Del Cesar	San Juan Del Cesar
Magdalena	Santa Bárbara De Pinto	Pivijay, Salamina, Ciénaga, Fundación, El Banco	Santa Bárbara De Pinto, Ciénaga	Pivijay, Ciénaga, Fundación	Ciénaga, Fundación	Salamina, Ciénaga, Fundación
Meta	Villavicencio, Acacías, Restrepo	Villavicencio, Acacías, El Castillo, Granada, Guamal, Puerto Concordia, Puerto López, Restrepo, San Martín	Villavicencio, Acacías, Barranca De Upía, Castilla La Nueva, Granada, Mesetas, Puerto López, Restrepo, San Juanito, San Martín, Mapiripán	Villavicencio, Acacías, Castilla La Nueva, Cubarral, Cumaral, Granada, Guamal, Lejanías, Puerto López, Puerto Lleras, Puerto Rico, San Martín, Vistahermosa	Villavicencio, Acacías, Barranca De Upía, Granada, Lejanías, Puerto López, Restrepo, San Martín, Vistahermosa	Villavicencio, Acacías, Puerto López, San Martín
Nariño	Pasto, Túquerres, Ipiales	Ricaurte, San Bernardo, San Pablo, Samaniego, Pasto, Albán, Buesaco, Colón, Nariño, Taminango, Tangua, Chachagüí, El Tablón De Gómez, Túquerres, Linares, Yacuanquer, La Cruz, El Tambo, Guachucal, La Unión, Leiva, Ipiales	El Rosario, Ricaurte, San Andrés De Tumaco, Sandoná, Potosí, San Pablo, Puerres, Pupiales, Samaniego, Pasto, Albán, Belén, Buesaco, Colón, Nariño, Taminango, Tangua, El Tablón De Gómez, Túquerres, El Tambo, Funes, La Unión, Leiva, Ipiales	Los Andes, Policarpa, Ricaurte, San Andrés De Tumaco, Sandoná, Puerres, Samaniego, Contadero, Pasto, Albán, Buesaco, Tangua, El Tablón De Gómez, Túquerres, Linares, El Tambo, La Unión, Gualmatán, Ipiales	Policarpa, Ricaurte, San Andrés De Tumaco, San Bernardo, Potosí, San Pablo, Puerres, Pasto, Albán, Cuaspud Carlosama, Buesaco, Colón, El Peñol, El Tablón De Gómez, Túquerres, Linares, La Unión, Ipiales	San Pedro De Cartago, Pasto, Buesaco, Funes, Ipiales

Norte De Santander	San José De Cúcuta	San José De Cúcuta, Ocaña, Pamplona	San José De Cúcuta, Ocaña, Pamplona, Sardinata, Teorama	San José De Cúcuta, Ábrego, Cáchira, Ocaña, Pamplona, Puerto Santander, Chitagá, Convención	San José De Cúcuta, Ábrego, La Playa, Ocaña, Pamplona, Convención	San José De Cúcuta, La Playa, Ocaña, Pamplona, Villa Del Rosario
Putumayo	Mocoa	Mocoa, Colón, Valle Del Guamuez, Villagarzón, Puerto Caicedo, Puerto Asís, Puerto Leguízamo	Mocoa, Colón, Orito, San Miguel, Valle Del Guamuez, Villagarzón, Puerto Asís, Puerto Leguízamo	Mocoa, Colón, Orito, San Miguel, Valle Del Guamuez, Villagarzón, Puerto Asís, Puerto Guzmán, Puerto Leguízamo	Mocoa, Colón, Orito, San Miguel, Valle Del Guamuez, Villagarzón, Puerto Asís, Puerto Guzmán, Puerto Leguízamo	Colón, Puerto Asís
Quindío	Buenavista, Calarcá, Córdoba, Montenegro	Armenia, Buenavista, Calarcá, Circasia, Córdoba, Filandia, Génova, La Tebaida, Montenegro, Quimbaya, Salento	Armenia, Buenavista, Calarcá, Circasia, Filandia, La Tebaida, Montenegro, Pijao, Quimbaya, Salento	Armenia, Calarcá, Circasia, Filandia, La Tebaida, Montenegro, Quimbaya	Armenia, Buenavista, Calarcá, Circasia, Filandia, Génova, La Tebaida, Montenegro, Pijao	Armenia, Córdoba, La Tebaida, Salento
Risaralda	Pereira, Dosquebradas, La Virginia	Pereira, Belén De Umbría, Dosquebradas, La Celia, La Virginia, Santa Rosa De Cabal, Santuario	Pereira, Apía, Balboa, Belén De Umbría, Dosquebradas, La Celia, La Virginia, Marsella, Mistrató, Pueblo Rico, Quinchía, Santa Rosa De Cabal, Santuario	Pereira, Apía, Belén De Umbría, Dosquebradas, Guática, La Virginia, Marsella, Mistrató, Santa Rosa De Cabal, Santuario	Pereira, Apía, Balboa, Belén De Umbría, Dosquebradas, Guática, La Celia, La Virginia, Marsella, Mistrató, Pueblo Rico, Quinchía, Santa Rosa De Cabal, Santuario	Pereira, Dosquebradas, La Celia, La Virginia
Santander	Floridablanca, Bucaramanga, San Gil	Zapatoca, Málaga, Floridablanca, Bucaramanga, Barrancabermeja, Charta, Chima, Rionegro, San José De Miranda, Vélez	Vetas, Málaga, Floridablanca, Bucaramanga, Barrancabermeja, Charalá, Rionegro, San Andrés, San Gil	El Playón, Floridablanca, Bucaramanga, Barrancabermeja, Charalá, Cimitarra	Zapatoca, La Paz, Málaga, Mogotes, Oiba, Contratación, Jesús María, Floridablanca, Bucaramanga, Barichara, Barrancabermeja, Charalá, Chima, San Gil, San Joaquín, Socorro, Suaita, Suratá, Valle De San José	Floridablanca, Bucaramanga, San Gil, San Miguel, Valle De San José
Sucre	Corozal	Sincelejo, Buenavista, Corozal, San Juan De Betulia, San Pedro, Santiago De Tolú	Sincelejo, Buenavista, Corozal, San Pedro, San Luis De Sincé	Sincelejo, Corozal	Sincelejo, Buenavista, Corozal, Palmito, San Juan De Betulia, San Pedro	Sincelejo, Corozal, Coveñas
Tolima	Ibagué, Coyaima, Lérida, San Sebastián De Mariquita	Ibagué, Anzoátegui, Armero, Cajamarca, Coyaima, Espinal,	Ibagué, Ambalema, Cajamarca, Casabianca,	Ibagué, Anzoátegui, Armero, Cajamarca, Carmen De Apicalá,	Ibagué, Alvarado, Anzoátegui, Armero, Cajamarca, Carmen	Ibagué, Chaparral, Espinal, Fresno, Lérida, Líbano,

		Falan, Fresno, Herveo, Lérida, Líbano, San Sebastián De Mariquita, Melgar, Planadas, Purificación, Roncesvalles, Rovira, San Antonio, Santa Isabel, Honda	Chaparral, Espinal, Falan, Fresno, Guamo, Herveo, Icononzo, Lérida, Líbano, San Sebastián De Mariquita, Melgar, Palocabildo, Planadas, Prado, Purificación, Roncesvalles, Rovira, San Antonio, Honda	Casabianca, Chaparral, Coello, Coyaima, Espinal, Falan, Fresno, Guamo, Lérida, Líbano, San Sebastián De Mariquita, Melgar, Planadas, Prado, Rovira, San Antonio, Valle De San Juan, Honda	De Apicalá, Chaparral, Coyaima, Espinal, Falan, Fresno, Guamo, Icononzo, Lérida, Líbano, San Sebastián De Mariquita, Melgar, Natagaima, Palocabildo, Piedras, Planadas, Prado, Purificación, Rovira, Saldaña, San Antonio, Venadillo, Villahermosa, Honda	Prado, Purificación, Honda
Valle Del Cauca	Cali, Florida, Palmira, Tuluá, Yumbo	Cali, Guadalajara De Buga, Candelaria, Cartago, El Cairo, El Dovio, Florida, Ginebra, Guacarí, Alcalá, Obando, Palmira, Roldanillo, San Pedro, Sevilla, Trujillo, Tuluá, Yumbo, La Victoria, La Cumbre, Dagua	Cali, Ansermanuevo, Argelia, Guadalajara De Buga, Caicedonia, Calima, Cartago, El Cairo, El Cerrito, Florida, Ginebra, Guacarí, Alcalá, Palmira, Restrepo, Roldanillo, Sevilla, Toro, Trujillo, Tuluá, Yumbo, Dagua	Cali, Argelia, Guadalajara De Buga, Caicedonia, Calima, Cartago, El Águila, El Cairo, El Dovio, Florida, Ginebra, Guacarí, Alcalá, Palmira, Roldanillo, Sevilla, Toro, Trujillo, Tuluá, Vijes, Yotoco, Yumbo, Zarzal, La Unión, Dagua	Cali, Ansermanuevo, Argelia, Guadalajara De Buga, Bugalagrande, Caicedonia, Calima, Cartago, El Cairo, El Dovio, Florida, Ginebra, Alcalá, Obando, Palmira, Riofrío, Roldanillo, Sevilla, Trujillo, Tuluá, Vijes, Yumbo, Bolívar, Dagua	Cali, Andalucía, El Cairo, Palmira, Sevilla, Tuluá
Vaupés	Taraira	None	Mitú, Carurú, Taraira	Mitú, Carurú	Mitú, Carurú, Taraira	Mitú
Vichada	None	None	Puerto Carreño	None	Santa Rosalía	None
